# Dosimetric advantages of daily adaptive strategy in IMPT for high‐risk prostate cancer

**DOI:** 10.1002/acm2.13531

**Published:** 2022-01-19

**Authors:** Hiroshi Tamura, Keiji Kobashi, Kentaro Nishioka, Takaaki Yoshimura, Takayuki Hashimoto, Shinichi Shimizu, Yoichi M. Ito, Yoshikazu Maeda, Makoto Sasaki, Kazutaka Yamamoto, Hiroyasu Tamamura, Hidefumi Aoyama, Hiroki Shirato

**Affiliations:** ^1^ Department of Radiation Oncology Graduate School of Biomedical Science and Engineering Hokkaido University Sapporo Japan; ^2^ Department of Radiological Technology Hokkaido University Hospital Sapporo Japan; ^3^ Department of Radiation Medical Science and Engineering Faculty of Medicine Hokkaido University Sapporo Japan; ^4^ Department of Medical Physics Hokkaido University Hospital Sapporo Japan; ^5^ Department of Health Sciences and Technology Faculty of Health Sciences Hokkaido University Sapporo Japan; ^6^ Data Science Center Promotion Unit Institute of Health Science Innovation for Medical Care Hokkaido University Hospital Sapporo Japan; ^7^ Proton Therapy Center Fukui Prefectural Hospital Fukui Japan; ^8^ Department of Radiation Oncology Faculty of Medicine Hokkaido University Sapporo Japan; ^9^ Global Center for Biomedical Science and Engineering Faculty of Medicine Hokkaido University Sapporo Japan

**Keywords:** adaptive therapy, intensity‐modulated proton therapy, prostate cancer, robust optimization

## Abstract

**Purpose:**

To evaluate the dosimetric advantages of daily adaptive radiotherapy (DART) in intensity‐modulated proton therapy (IMPT) for high‐risk prostate cancer by comparing estimated doses of the conventional non‐adaptive radiotherapy (NART) that irradiates according to an original treatment plan through the entire treatment and the DART that uses an adaptive treatment plan generated by using daily CT images acquired before each treatment.

**Methods:**

Twenty‐three patients with prostate cancer were included. A treatment plan with 63 Gy (relative biological effectiveness (RBE)) in 21 fractions was generated using treatment planning computed tomography (CT) images assuming that all patients had high‐risk prostate cancer for which the clinical target volume (CTV) needs to include prostate and the seminal vesicle (SV) in our treatment protocol. Twenty‐one adaptive treatment plans for each patient (total 483 data sets) were generated using daily CT images, and dose distributions were calculated. Using a 3 mm set‐up uncertainty in the robust optimization, the doses to the CTV, prostate, SV, rectum, and bladder were compared.

**Results:**

Estimated accumulated doses of NART and DART in the 23 patients were 60.81 ± 3.47 Gy (RBE) and 63.24 ± 1.04 Gy (RBE) for CTV D99 (*p* < 0.01), 62.99 ± 1.28 Gy (RBE) and 63.43 ± 1.33 Gy (RBE) for the prostate D99 (*p* = 0.2529), and 59.07 ± 5.19 Gy (RBE) and 63.17 ± 1.04 Gy (RBE) for SV D99 (*p* < 0.001). No significant differences were observed between NART and DART in the estimated accumulated dose for the rectum and bladder.

**Conclusion:**

Compared with the NART, DART was shown to be a useful approach that can maintain the dose coverage to the target without increasing the dose to the organs at risk (OAR) using the 3 mm set‐up uncertainty in the robust optimization in patients with high‐risk prostate cancer.

## 1 INTRODUCTION

In recent years, intensity‐modulated proton therapy (IMPT), a new irradiation technique for prostate cancer, has been used to create a highly flexible dose distribution by optimizing the beam direction and energy fluence using the proton beam specific Bragg peak. In principle, this technique makes it possible to reduce toxicity to the patient by concentrating the dose to the target while reducing the dose to the surrounding organs at risk (OAR).^1^, ^2^, ^3^, ^4^

Currently the original treatment plan generated based on the planning computed tomography (CT) images acquired more than a week before the start of treatment was mostly used for the entire treatment by assuming that the shape of prostate and seminal vesicle (SV) as the treatment target and the rectum and bladder as OAR remain unchanged. However, the patient anatomy including prostate rotation, bladder volume, and rectal shape and other specifics may undergo a variety of changes during the treatment period (about 1–2 months for the prostate cancer treatment) from the time of the planning CT.^5^, ^6^, ^7^, ^8^ It has been pointed out that these anatomical changes may cause discrepancies between the dose distribution by the original treatment plan and the actual dose distribution, resulting in insufficient doses to the target or unexpectedly high doses to the OAR.^4^, ^9^, ^10^, ^11^, ^12^, ^13^, ^14^, ^15^, ^16^, ^17^, ^18^, ^19^ In the IMPT treatment technique, the ideal dose distribution can be generated to reduce the rectum dose located dorsal to the prostate and SV without reducing the target coverage in comparison to that with the conventional technique, and thus the non‐uniformed dose distribution of each field tends to have a steep gradient, which makes treatment planning sensitive to any uncertainties such as daily anatomical changes.^20^, ^21^, ^22^, ^23^, ^24^ Distortion of the actual dose distribution due to the daily anatomical changes may make it difficult to deliver the planned dose accurately and so reduce the essential advantages of IMPT.

Recently, Goupy et al.^25^ have suggested that the proportion of patients diagnosed with high‐risk prostate cancer invading the SV is increasing due to the generalization of multiparametric magnetic resonance imaging. They showed the difficulty to give a sufficiently high dose to the SV without increasing the adverse effects using intensity‐modulated X‐ray therapy. In IMPT, Thörnqvist et al.^15^ reported that the estimated actual dose recalculated from eight to nine repeat CT images in patients showed a significant decrease for SV D99 and a generalized equivalent uniform dose compared to the planned dose, which could not be resolved by expanding the margin or using image guided radiotherapy. For passively scattered proton therapy treatment plans, Maeda et al.^18^ estimated the actual dose from a total of 193 fractions of high‐risk patients including the SV in the clinical target volume (CTV) and found that less than 40% of the CTV V95 (%) maintained the value of the original planned dose. They suggested that the daily adaptive radiotherapy (DART) approach using an in‐room CT could be a solution to spare the rectum while maintaining the dose to the SV in the high‐risk group of patients.^18^

Research on the adaptive strategy has been actively investigating photon and proton therapy.^26^, ^27^, ^28^, ^29^, ^30^, ^31^, ^32^ However, there is no report that directly compares the estimated actual dose and the planned dose using complete sets of daily in‐room CT images in IMPT, as far as we are aware. To the best of our knowledge, this is the first study to compare the dosimetric effect of the two treatment strategies in prostate IMPT: non‐adaptive radiotherapy (NART) with only the original treatment plan and DART with adaptive treatment plans using high‐quality CT images for patients acquired before every treatment.

In the proton beam community, a 5 mm set‐up uncertainty has been widely used in plans of robust optimization for IMPT. On the other hand, Xu et al.^16^ have recently reported that a 3 mm set‐up uncertainty was sufficient in the robust optimization for NART in 10 patients using cone beam CT. However, they have also shown that SV was outside the CTV coverage in some patients. Our working hypothesis in this study was that a 3 mm set‐up uncertainty is appropriate in DART but insufficient in NART for high‐risk prostate cancers for which CTV needs to include both the prostate and SV. Therefore, in this study, the same 3 mm set‐up uncertainty was retrospectively used both in NART and in DART to investigate whether the coverage for CTV would be improved with the usage of DART assuming that all patients had high‐risk prostate cancer.

## 2 METHODS

### 2.1 Patient data

This study used the original planning CT images and the daily in‐room CT images for 23 patients who underwent the prostate cancer treatment at the Fukui Prefectural Hospital Proton Therapy Center. Daily in‐room CT images were acquired by a self‐propelled CT scanner on rails (Aquilion LB, Toshiba Medical Systems, Tochigi, Japan). The number of daily CT sets acquired per patient was 36–40, with 21 consecutive sets from the first treatment session used in accordance with the protocol for this study. A total of 483 sets of daily CT were used in the analysis. This study was approved by the institutional review board.

All patients were immobilized in the supine position using a suction‐type fixed bag and a thermoplastic shell (RSF‐19Gl and ESS‐25, ESFORM; Engineering System Co., Ltd., Nagano, Japan). The bladder volume was as full as possible at the time of the planning CT and daily CT. The daily bladder volume was monitored by ultrasound scans to ensure that the volume was similar to that in the planning CT, and patients were instructed to drink water when the bladder was not sufficiently distended. The planning CT scan protocol was 120 kV, 480 mA, and the daily CT scan protocol 120 kV, 150 mA. The CT images were reconstructed with a slice thickness of 2 mm and a transversal pixel size of 1.07 × 1.07 mm^2^.^18^

### 2.2 Treatment planning simulation

Experienced radiation oncologists created regions of interest (ROI) for the prostate, SV, rectum (from the rectosigmoid flexure to the anus), and bladder on the planning CT images. Also, we created ROI for all sets of the daily CT images for the prostate, SV, rectum, and bladder using the deformed image registration (DIR) function of the treatment planning support software MIM version 6.9 (MIM Software Inc., Cleveland, OH, USA) with ROI on planning CT images as a reference. Then experienced radiation oncologists modified this if needed.^18^, ^19^, ^33^ In several daily CT images, the upper part of the bladder was a slightly absent due to insufficient imaging area. Even then, since the same CT images were used both for NART and DART and the high‐dose region was examined in all patients, a comparative study was also performed for the bladder. We delineated the bladder's ROI limited to the area observed in the daily CT images and used this for the analysis in this study.

A treatment planning system VQA (Hitachi Ltd., Hitachi, Japan) was used for the treatment planning, and the prescribed dose was 63 Gy (relative biological effectiveness (RBE)) in 21 fractions by two opposed lateral field IMPT with multi‐field optimization (MFO). The dose was calculated using a 2 mm isotropic dose grid, and the RBE was kept constant at 1.1.

Assuming that all the patients had high‐risk prostate cancer, the CTV including both the prostate and the entire SV was retrospectively delineated for all patients for the purposes of this study. The planning dose objectives were set to CTV D99 (Gy (RBE)) >100% and Dmax (Gy (RBE)) <110% of the prescribed dose (Table 1), where D99 represents the minimum dose to 99% of the target volume. In IMPT treatment planning, simply increasing the geometric margin to the target may not ensure a sufficient dose due to unpredictable changes in the patient anatomy during the treatment.^34^, ^35^ Therefore, we did not set a margin for the CTV, but set a robust optimization using the worst case optimization as proposed by Pflugfelder et al.^34^ to account for uncertainties throughout the course of the treatment at 3 mm (set‐up uncertainty related to the inter‐ and intra‐fractional motion) and 3.5% (range uncertainty related to the patient stopping power ratio estimation). The OAR applied the following dose constraints: V50 < 20% (as low as possible for severe cases), V30 < 50% for the rectum, and V30 < 30% for the bladder (Table 1), where V50 and V30 represent the relative volume receiving at least 50 Gy (RBE) and 30 Gy (RBE).

**TABLE 1:** Mean and standard deviation (SD) for the dose volume histogram (DVH) indices of the original treatment plan in the 23 patients

DVH indices	Planning goal	Mean	SD	Rate of achieving goal
CTV D99	>63 Gy (RBE)	63.5 Gy (RBE)	0.2 Gy (RBE)	100.0%
CTV Dmax	<69.3 Gy (RBE)	67.4 Gy (RBE)	1.0 Gy (RBE)	100.0%
Prostate D99	n/a	63.5 Gy (RBE)	0.3 Gy (RBE)	n/a
SV D99	n/a	63.4 Gy (RBE)	0.2 Gy (RBE)	n/a
Rectum Dmax	n/a	65.4 Gy (RBE)	0.8 Gy (RBE)	n/a
Rectum V30	<50%	32.1%	7.1%	100.0%
Rectum V50	<20%	17.0%	3.5%	87.0%
Rectum V60	n/a	7.5%	1.9%	n/a
Bladder V30	<30%	18.2%	7.5%	95.7%
Bladder V60	n/a	4.6%	2.6%	n/a
Bladder V63	n/a	4.1 ml	2.0 ml	n/a

Abbreviations: CTV, clinical target volume; RBE, relative biological effectiveness; SV, seminal vesicle.

To evaluate the robustness of the original treatment plan for the target and OAR, the plans under the error scenarios with shifted isocenter in the original treatment plan at 3 mm in six directions (right, left, head, foot, anterior, and posterior) and with varied Hounsfield Unit (HU) on the planning CT at ±3.5% were created voxel by voxel. Then, the dose volume histogram (DVH) indices of the plans under the error scenarios and the original treatment plan were compared. The time required for dose calculation and optimization for all original and adaptive treatment plans was recorded.

### 2.3 Delivered estimated dose recalculation assuming the NART and DART strategies

To compare the two treatment strategies, we recalculated the daily estimated dose assuming (1) NART: irradiation with the original treatment plan generated using planning CT in all fractionation schedules and (2) DART: irradiation with the adaptive treatment plan generated using daily CT in all fractionation schedules.

For the dose recalculations using the total 483 sets of daily CT, we used an in‐house dose calculation program (DCP) which implements the same calculation algorithm as the VQA used for treatment planning, to automate the process of performing the calculations and for an efficient analysis of the DVH indices. To confirm the accuracy of the DCP, several treatment plans were recalculated by VQA and the DCP using a digital water phantom. A three‐dimensional (3D) gamma analysis was performed using MIM to evaluate the differences between the dose distributions calculated by VQA and DCP. All original and adaptive digital imaging communication in medicine (DICOM) data (CT, ROI, Dose, Plan) generated by VQA were transferred to the DCP.

Logically, it would be sensible to use only the prostate for the alignment but slight differences in daily manual prostate contouring can cause large differences in the rotational alignment. To control for this, we first corrected the rotational alignment error with bone structure matching in six axes using the rigid image registration (RIR) function of MIM. This process is also useful to reduce the uncertainties in the beam path length due to daily changes of the bone structures. After the RIR, the positional deviation of the prostate, SV, rectum, and bladder relative to the bone structures were visually assessed using sagittal and axial images. Following this, the geometric center of the prostate in the daily CT was registered to the isocenter of the planning CT.

**FIGURE 1:**
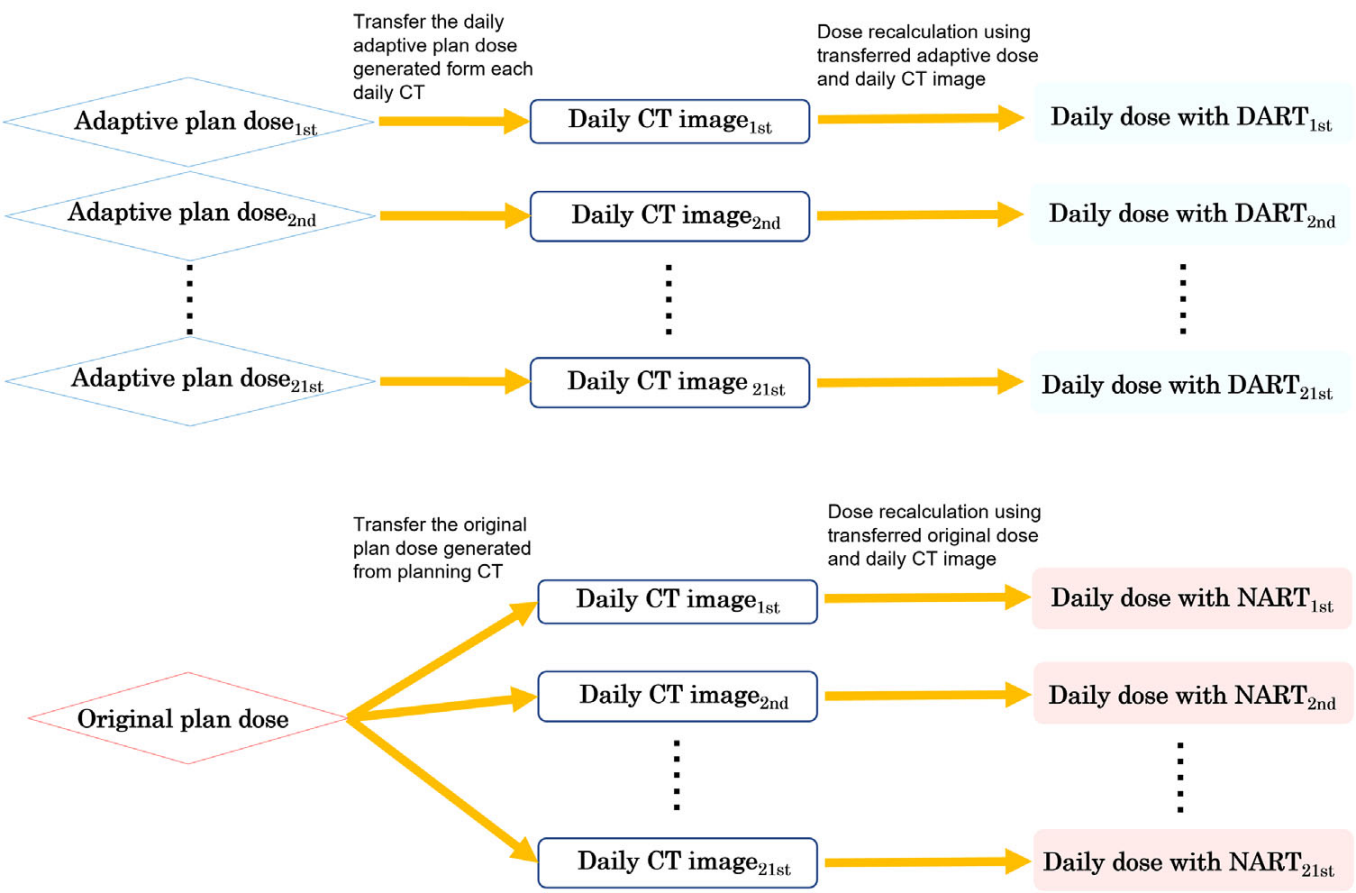
Simulated dose recalculation for the non‐adaptive radiotherapy (NART) and daily adaptive radiotherapy (DART) strategies in dose calculation program (DCP). The NART and DART dose distributions for 21 fractions per patient were recalculated. Original plans were applied to daily computed tomography (CT) for NART, and daily adaptive plans were applied to daily CT for DART

Simulation of the treatment strategy was performed in DCP as shown in Figure 1. The dose calculation on each daily CT was performed based on the original treatment plan and the daily adaptive plan for NART and DART, respectively.

### 2.4 Analysis of the DVH indices

The DVH indices were used to compare the fractional and accumulated dose distributions recalculated assuming the NART and DART strategies. The prostate D99 (Gy (RBE)), SV D99 (Gy (RBE)), and CTV D99 (Gy (RBE)) for target coverage and rectum V30 (%), rectum V50 (%), rectum V60 (%), bladder V30 (%), bladder V60 (%), and rectum Dmax (Gy (RBE)) were selected as the evaluation indices. The volume in milliliters of bladder which received 63 Gy or more (V63 (ml)), which is not affected by the field limitations of the viewed images, was also evaluated in this study. In the evaluations of the fractional dose distribution, each fractionated dose was scaled to 21 times to investigate the dose distribution in the worst case scenario. In the evaluation of the accumulated dose distribution, the true dose was calculated by summing each fraction dose together. The DIR algorithm in the MIM was used to estimate the accumulated dose through the entire treatment both in NART and in DART. Each of the 21 fractionated dose distributions on the daily CT images was deformed into the dose distribution of the planning CT images for each patient. Finally, we calculated the estimated accumulated dose distribution by adding the deformed 21 daily dose distributions on the same planning CT.

### 2.5 Statistical analysis

The JMP PRO version15.2 (SAS Institute, Cary, NC, USA) was used for the statistical analysis. Paired *t*‐tests were used for the statistical significance of the DVH indices between the NART and DART strategies. McNemar's test was used for the statistical significance of the rates of achieving clinical goals (Table 2) for the DVH indices between the NART and DART strategies with the *p*‐value = 0.05 as the significance level.

**TABLE 2:** Comparison of rates of achieving clinical goals between non‐adaptive radiotherapy (NART) and daily adaptive radiotherapy (DART) in the fractional and accumulated doses

		Fractional dose (*N* = 483)			Accumulated dose (*N* = 23)		
DVH indices	Clinical goal	NART	DART	*p*‐Value	NART	DART	*p*‐Value
CTV D99	>59.85 Gy (RBE)	56.5%	100.0%	<0.001	82.6 %	100.0%	<0.05
Prostate D99	>59.85 Gy (RBE)	98.6%	100.0%	<0.01	95.7%	95.7%	1.000
SV D99	>59.85 Gy (RBE)	37.9%	100.0%	<0.001	47.8%	95.7%	<0.01
Rectum Dmax	<66 Gy (RBE)	53.0%	85.7%	<0.001	95.7%	100.0%	0.3173
Rectum V30	<50%	95.0%	99.2%	<0.001	95.7%	100.0%	0.3173
Rectum V50	<20%	69.2%	89.2%	<0.001	69.6%	87.0%	0.1025
Bladder V30	<30%	99.0%	99.8%	<0.05	91.3%	91.3%	1.000
Bladder V63	<10 ml	96.5%	98.1%	<0.001	100.0%	100.0%	1.000

Abbreviations: CTV, clinical target volume; DVH, dose volume histogram; RBE, relative biological effectiveness; SV, seminal vesicle.

## 3 RESULTS

### 3.1 Anatomical changes through the entire treatment

**FIGURE 2:**
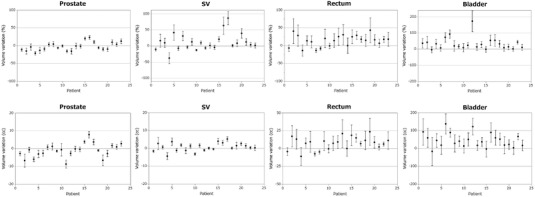
The volume differences between planning computed tomography (CT) and daily CT for the prostate, seminal vesicle (SV), rectum, and bladder in the 21 fractions for each of the 23 patients. Dots and error bars represent the means and standard deviations in the 21 fractions

Figure 2 shows the means and standard deviations (SD) of the volume differences between planning CT and daily CT for the prostate, SV, rectum, and bladder in the 21 fractions for each of the 23 patients. The volume differences in percentages between the planning CT and daily CT in all 483 sets were ‐2.13 ± 12.88%, 12.18 ± 28.88%, 14.54 ± 24.54%, and 34.10 ± 46.22% for the prostate, SV, rectum, and bladder. The values for the SV, rectum, and bladder showed greater differences than those for the prostate. In 71% and 82% of 483 sets of daily CT, the volumes of the rectum and bladder were larger than the planning CT.

### 3.2 DVH indices of the original treatment plan

The average time required for dose calculation and optimization for the original treatment plan in this study was 22.1 min in 23 patients. Table 1 shows the means and SD of the DVH indices for the CTV, prostate, SV, rectum, and bladder in the original treatment plan generated by 23 sets of planning CT. Dose objectives of the target were achieved in all patients as the target dose objective was prioritized over the OAR dose constraints. The OAR dose constraints were achieved in all patients for rectum V30 (%), in 20 of 23 patients for rectum V50 (%), and in 22 of 23 patients for bladder V30 (%).

The differences between the plans under the error scenarios and the original treatment plan were ‐0.54 ± 0.46 Gy (RBE) for CTV D99, 0.31 ± 3.21% for rectum V60, and ‐0.04 ± 2.01% for bladder V60.

### 3.3 Direct comparison of the estimated actual dose with the NART and DART strategies

The results of a 3D gamma analysis comparing the dose distributions calculated by VQA and DCP were 99.96 ± 0.023% on the pass rate of 1 mm and 1% criteria, showing that good agreement was achieved.

**FIGURE 3:**
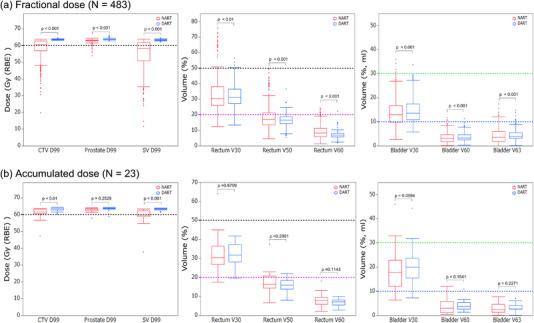
Boxplots comparing estimated actual doses recalculated using the 483 sets of daily computed tomography (CT) with non‐adaptive radiotherapy (NART) (red) and daily adaptive radiotherapy (DART) (blue) treatment strategies for fractional doses (a) and accumulated doses (b). For the target, the black dashed line represents the clinical goals for CTV D99 (Gy (RBE)), prostate D99 (Gy (RBE)), and SV D99 (Gy (RBE)). For the organs at risk (OAR), the black dashed line represents the clinical goals for rectum V30 (%), the magenta dashed line for rectum V50 (%), the light green dashed line for bladder V30 (%), and the blue line for bladder V63 (ml)

Figure 3a shows the comprehensive comparison of the estimated fractional doses (483 sets) to the target and OAR when irradiated with the NART and DART strategies. The mean estimated fractional doses of DART were higher than those of NART with statistical significance for CTV D99 (Gy (RBE)), prostate D99 (Gy (RBE)), and SV D99 (Gy (RBE)). For the OAR, the mean estimated fractional doses of DART were lower than those of NART with statistical significance for rectum V30 (%), rectum V50 (%), rectum V60 (%), and rectum Dmax (Gy (RBE)). With the mean estimated fractional doses of bladder V30 (%), bladder V60 (%), and bladder V63 (ml) of DART were higher than those of NART with statistical significance.

Figure 3b shows the comprehensive comparison of the estimated accumulated doses (23 patients) to the target and OAR when irradiated with the NART and DART strategies. The means and SD of the estimated accumulated doses of the NART and DART in the 23 patients were 60.81 ± 3.47 Gy (RBE) and 63.24 ± 1.04 Gy (RBE) for CTV D99 (*p* < 0.01), 62.99 ± 1.28 Gy (RBE) and 63.43 ± 1.33 Gy (RBE) for the prostate D99 (*p* = 0.2529), and 59.07 ± 5.19 Gy (RBE) and 63.17 ± 1.04 Gy (RBE) for SV D99 (*p* < 0.001). There were no statistically significant differences between NART and DART in any of the DVH indices for rectum and bladder.

**FIGURE 4:**
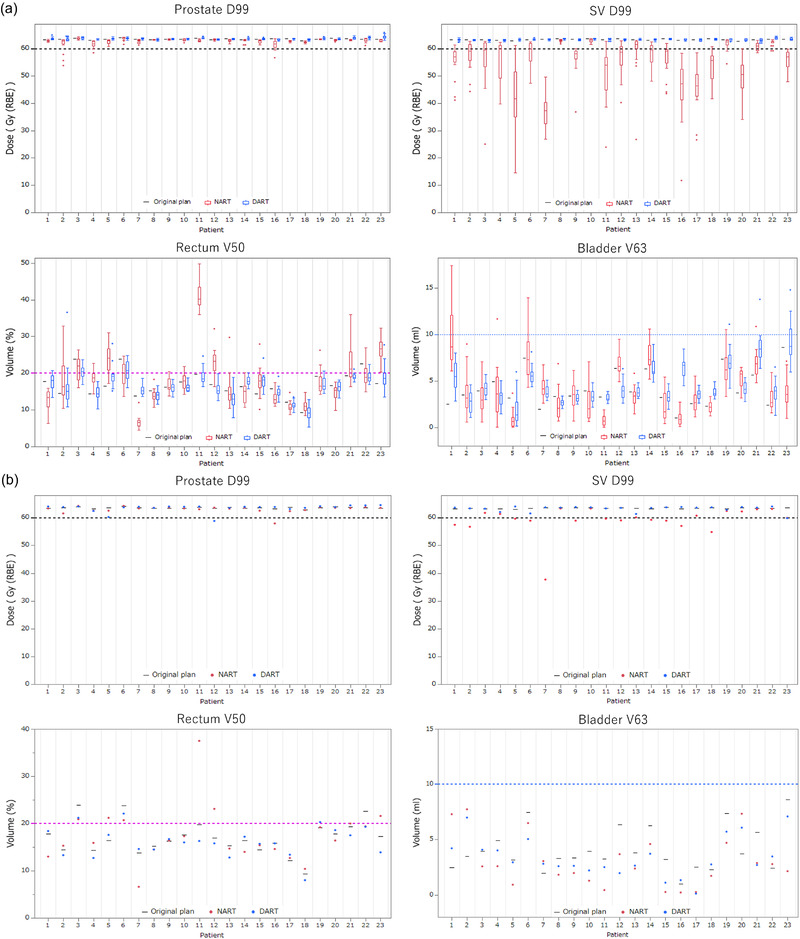
Comparing estimated fractional doses (a) and accumulated doses (b) for each patient of prostate D99 (Gy (RBE)), SV D99 (Gy (RBE)), rectum V50 (%), and bladder V63 (ml) with the non‐adaptive radiotherapy (NART) (red) and daily adaptive radiotherapy (DART) (blue) treatment strategies through the entire treatment. Black lines represent the dose volume histogram (DVH) indices for each patient in the original treatment plan, and the dashed lines represent the clinical goals for each DVH indices

Figure 4 shows the comparison of the estimated (a) fractional doses and (b) accumulated doses for each patient of prostate D99 (Gy (RBE)), SV D99 (Gy (RBE)), rectum V50 (%), and bladder V63 (ml) for the NART and DART strategies. No significant dose reduction of prostate D99 (Gy (RBE)) occurred with NART and DART. However, there were unacceptable dose reductions for SV D99 (Gy (RBE)) with NART especially in the fractional doses. In patients 5, 11, 12, and 23, rectum V50 (%) was higher than the clinical goal with NART, but those with DART could be assigned as lower than the clinical goals. Bladder V63 (ml) was mostly lower than the clinical goal with NART and DART in all patients.

### 3.4 Rates of achieving clinical goals of the DVH indices

Table 2 shows the results of the rates of achieving the clinical goals of the NART and DART strategies in the fractional and accumulated dose distributions. The clinical goals for CTV D99 (Gy (RBE)), prostate D99 (Gy (RBE)), and SV D99 (Gy (RBE)) are defined as 95% of the prescribed dose.

In the fractional dose estimates, the NART achieved the clinical goal in 56.5% of all sets for CTV D99 (Gy (RBE)), 98.6% for prostate D99 (Gy (RBE)), and 37.9% for SV D99 (Gy (RBE)). The significantly lower percentage for SV D99 (Gy (RBE)) is noteworthy. However, the DART achieved the clinical goal for all sets of CTV D99 (Gy (RBE)), prostate D99 (Gy (RBE)), and SV D99 (Gy (RBE)). For OAR, the NART and DART achieved the clinical goals in 95.0% and 99.2% of all sets for rectum V30 (%), 69.2% and 89.2% for rectum V50 (%), 53.0% and 85.7% for rectum Dmax (Gy (RBE)), 99.0% and 99.8% for bladder V30 (%), and 96.5% and 98.1% for bladder V63 (ml), and in all of these NART and DART were significantly different. In three patients who did not achieve the dose constraint for rectum V50 (%) in the original treatment plan, the rates of achieving clinical goals for rectum V50 (%) with NART and DART were 49.2% and 52.4% (*p* = 0.6949). There was no significant difference in the rates of achieving clinical goals for rectum V50 (%) of the NART and DART in the three patients who did not achieve the dose constraint for rectum V50 (%) in the original treatment plan. In 20 patients who achieved the dose constraint for rectum V50 (%) in the original treatment plan, the rates of achieving clinical goals of the NART and DART were 72.1% and 94.8% (*p* < 0.001).

In the accumulated dose estimates, statistically significant differences remain for CTV D99 (Gy (RBE)) and SV D99 (Gy (RBE)) but not for the others. Two patients out of the three who did not achieve the dose constraint for rectum V50 (%) in the original treatment plan did not achieve the clinical goals even with DART.

### 3.5 Comparison of the estimated dose distributions

**FIGURE 5:**
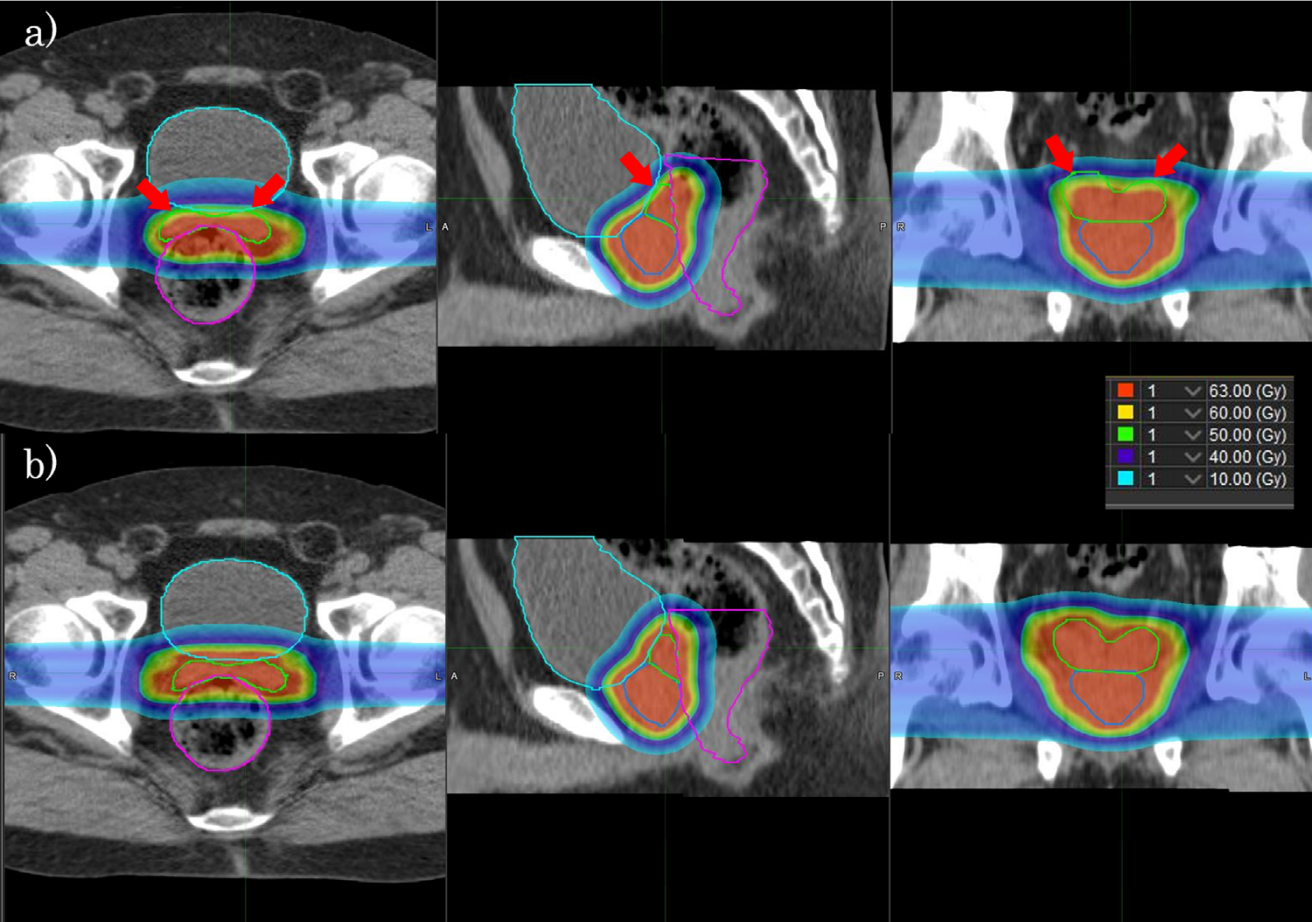
Representative example of the estimated actual dose distributions of non‐adaptive radiotherapy (NART) (a) and daily adaptive radiotherapy (DART) (b) using the same daily computed tomography (CT) images in the axial, sagittal, and coronal planes. The regions of interest (ROI) are displayed in the prostate (blue), seminal vesicle (SV) (light green), rectum (magenta), and bladder (light blue). The notable difference between two treatment strategies is failing to cover the anterior cranial side of SV in NART

**FIGURE 6:**
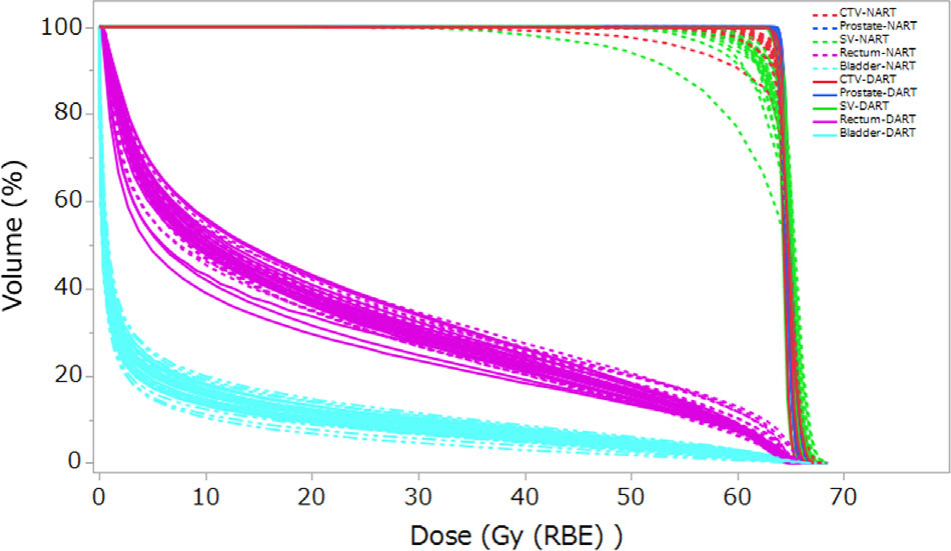
Comparison of the estimated actual dose volume histogram (DVH) of non‐adaptive radiotherapy (NART) (dashed lines) and daily adaptive radiotherapy (DART) (solid lines) recalculated for 21 fractions of the same patient as shown in Figure 5. Colors are displayed for the clinical target volume (CTV) (red), prostate (blue), seminal vesicle (SV) (light green), rectum (magenta), and bladder (light blue)

Figure 5 shows the dose distributions of NART and DART using the same set of daily CT for a representative case (patient 9). Figure 6 shows a comparison of the estimated actual DVH for all 21 fractions in the same patient. Comparison of the dose distribution and DVH for the two treatment strategies shows that NART does not adequately cover the dose to the SV, but DART improves on that. The dose to the rectum of NART tended higher than that of DART in the high‐dose area, and the dose to the bladder of NART was slightly lower than that of DART.

## 4 DISCUSSION

Spot optimization for IMPT treatment planning includes single‐field optimization (SFO) and MFO, which have different sensitivities to any uncertainties. The MFO‐IMPT provides a better dose distribution than SFO‐IMPT but is more sensitive to any uncertainties due to its more complex dose distribution.^36^ In this study, we simulated irradiation of MFO‐IMPT for prostate cancer with daily anatomical changes using two treatment strategies: NART with the original treatment plan and DART with the adaptive treatment plan generated using daily CT, and we evaluated the potential dose advantage of DART by comparing the target coverage and OAR sparing.

**FIGURE 7:**
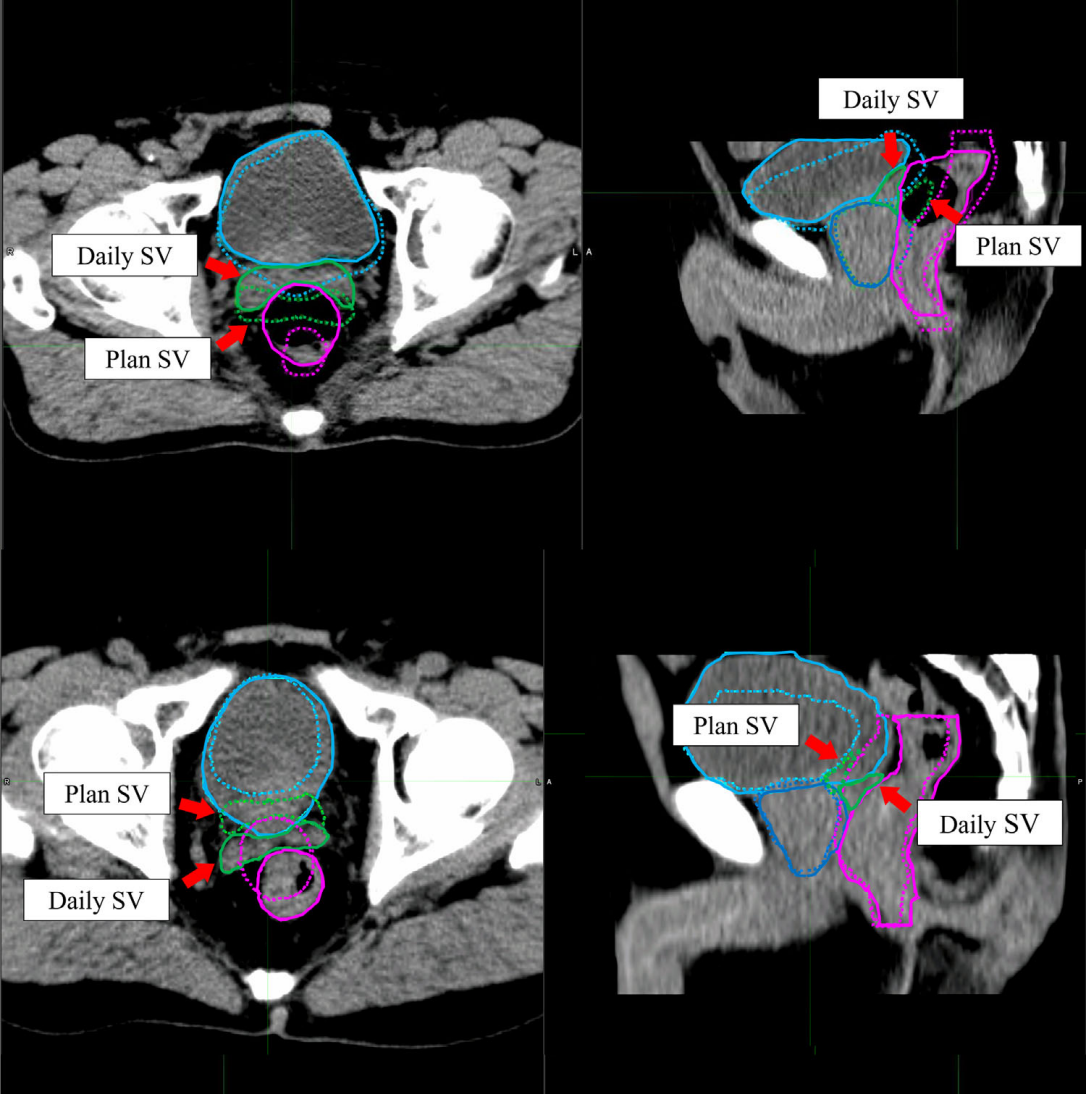
Inter‐fractional seminal vesicle (SV) positional deviations due to the rectal and bladder volume variations are shown. The upper and lower panels show the volume variations of rectum and bladder, respectively. The daily regions of interest (ROI) (solid line) and the original plan ROI (dashed line) are shown on the daily computed tomography (CT) image in the prostate (blue), seminal vesicle (SV) (light green), rectum (magenta), and bladder (light blue)

For the NART strategy, the mean estimated fractional dose for prostate D99 (Gy (RBE)) had a slight decrease from the prescribed dose, but that for SV D99 (Gy (RBE)) was about 13% lower than the prescribe dose. Because CTV was defined in the prostate plus the entire SV assuming a high‐risk group, this also resulted in a decrease of the mean estimated actual dose for CTV D99 (Gy (RBE)). The most likely reason for this is that the position of the SV was pushed forward in many cases due to the increased rectal volume considering that the volumes of the rectum were larger than the planning CT in 71% of the daily CT as shown in Figure 7. In other cases, the SV was pushed backward due to the posterior expansion of the bladder, resulting in a decrease of SV D99 (Gy (RBE)) as shown in Figure 7. In a detailed study of SV positional changes, Mak et al.^37^ showed that there is a strong correlation between rectal volume in the area adjacent to the SV and inter‐fractional SV positional deviation in the anterior–posterior direction. They also showed that the posterior point of the bladder was strongly correlated with the anterior–posterior movement of SV, so it is possible that SV D99 decreased as a result of the SV being pushed backward by the bladder expanding posteriorly in some patients. Frank et al.^38^ have shown that the margin for inter‐fractional change should be larger for SV than for the prostate.

In the present study, the original plans were evaluated as sufficiently robust since the changes in the plans under the error scenarios relative to the original plan was ‐0.54 ± 0.46 Gy (RBE) for CTV D99. However, the results for CTV D99 (Gy (RBE)) and SV D99 (Gy (RBE)) in accumulated dose estimates suggest that robust optimization with a 3 mm set‐up uncertainty in the plan is too small for CTV with NART. The present study suggests that an uncertainty larger than 3 mm is required in NART, but there are concerns about higher doses to the OAR. The dosimetric effect by expanding the uncertainty in NART will be the theme of a future study. Thörnqvist et al.^15^ evaluated the estimated actual doses recalculated using eight to nine repeat CT of each patient for IMPT with margins of up to 10 mm for the prostate, SV, and pelvic lymph nodes. The average decrease in SV D99 from the original treatment plan was 13%, even when set up with a reference marker implanted in the prostate, suggesting that simple margin expansion is not a solution for dose coverage of the SV.^15^ The present study compares the estimated actual doses of NART and DART and suggests that DART using the 3 mm for the set‐up uncertainty may offer a solution to improve the dose coverage of the SV in the modest fractionation schedule (21 times). Considering the large differences in the fractional dose estimates in this study, the DART strategy is mandatory if we use the recently reported ultra‐hypofractionated schedule.^39^

A direct comparison of the estimated fractional dose of NART and DART showed that DART achieved significantly lower rectum V30 (%), V50 (%), V60 (%), and Dmax (Gy (RBE)). Furthermore, the clinical goal achievement rate of DART was significantly higher, resulting in DART as also effective in reducing the dose to the rectum. However, even in DART, the dose constraint of rectum V50 (%) was not achieved in 52 sets (10.8%). About 60% of these occurred in three patients who did not achieve the dose constraint for the rectum V50 (%) in the original treatment plan. Two out of these three patients also failed to achieve the clinical goal with DART in the accumulated dose. This indicates that for patients with unique anatomies such as with the proximity of the SV to the rectum makes it difficult to maintain the dose to the SV and achieve the dose constraint of the rectum in the original treatment plan, and it is unlikely that the dose constraint of the rectum can be achieved even with DART.

A direct comparison of the estimated fractional dose for bladder V30 (%), V60 (%), and V63 (ml) between NART and DART showed that NART achieved significantly lower values and no statistically significant differences in the accumulated dose. Looking at the clinical goal achievement rate, DART achieved significantly higher rates in the fractional dose. The contradictory results about the mean estimated fractional doses of bladder V30 (%), V60 (%), and V63 (ml) suggest that insufficient coverage in the daily CT images may have affected the accuracy of the results for the bladder in this study. Since DART had a higher clinical goal achievement rate in the fractional dose and no statistically significant difference in the accumulated dose, we think that this result is not clinically relevant and does not preclude the superiority of DART.

The adaptive strategy in this study assumed that the adaptive plan was generated and irradiation was based on only the daily CT taken before the irradiation, and real‐time adaptation for intra‐fractional motion by real‐time monitoring, for example, using magnetic resonance imaging, was not used during the actual irradiation. Instead of the real‐time adaptation, the 3 mm set‐up uncertainty accounting for intra‐fractional motion was used in the robust optimization of adaptive plans for DART. Tang et al.^40^ estimated the actual dose considering intra‐fractional motion from Calypso tracking data of only the prostate movement during IMPT irradiation and investigated the dose differences from the original treatment plan. They used the prostate plus 1 cm of the proximal SV as CTV and reported that the reduction of CTV D99 compared with the original treatment plan was less than 2% on average through the entire treatment. However, Gill et al.^41^ measured intra‐fractional motion of the prostate and SV using cinematic magnetic resonance imaging and found no correlation between prostate and SV deviation. A real‐time adaptive strategy that also monitors and adapts for intra‐fractional motion of SV needs to be investigated in the future.

Table 1 shows the dose in the original treatment plan, in which 13% and 4.3% of the plan failed to meet the respective rectum V50 (%) and bladder V30 (%) goals. It is notable that in DART, both improved with respectively 10.8% and 0.2% in the fractional dose estimates. These differences cannot be explained by technological reasons and may be because the patients had better bladder and rectum preparation during the treatment than before the planning CT simulation.

A limitation on implementing the DART strategy is that the average time required for dose calculation and optimization for the daily adaptive plan in the 483 sets was as long as 22.5 min. Gill et al.^41^ pointed out that prostate and SV movements increase over time and reach the maximum displacement after 10 min. The longer it takes to generate the plan, the larger the movement of the prostate and SV will be, so the time required for adaptive planning should be as short as possible. A recent study reported that auto‐segmentation using deep learning can reduce the time required for contouring.^42^ Further, Matter et al.^43^ reported that by implementing graphic processing units (GPU) in the online adaptation planning process, a typical IMPT treatment plan could be generated in 5–10 s. As presented above, we align the bone structure first for rotational errors with bone matching in six axes, then align to the geometric center of the prostate. If the daily manual prostate contouring is improved by state‐of‐art technology, we may become able to skip the bone matching. Speeding up the adaptation process in this study is an issue for the future.

## 5 CONCLUSIONS

Compared with the conventional NART method which significantly reduces the dose to the SV, DART presents significant dosimetric advantages in maintaining dose coverage to the target without increasing the dose to the OAR. We suggest that if a more speedy adaptive planning is achieved, DART will become an attractive strategy in future IMPT for high‐risk prostate cancer.

## CONFLICT OF INTEREST

The authors have no conflicts of interest to declare.

## AUTHOR CONTRIBUTIONS

Hiroshi Tamura and Yoshikazu Maeda collected the data and made the simulation plans. Hiroshi Tamura and Keiji Kobashi analyzed and interpreted the data. Hiroshi Tamura, Kentaro Nishioka, Takayuki Hashimoto, Keiji Kobashi, Takaaki Yoshimura, and Hiroki Shirato wrote the draft of this paper. Keiji Kobashi, Shinichi Shimizu, Makoto Sasaki, Kazutaka Yamamoto, Hiroyasu Tamamura, Hidefumi Aoyama, and Hiroki Shirato supervised the project. Yoichi M. Ito participated in the statistical analysis. All authors read and approved the final manuscript.
